# Supplementary material for: Modeling glioblastoma heterogeneity as a dynamic network of cell states
Source: Mol Syst Biol. 2021 Sep 16;17(9):e10105. doi: 10.15252/msb.202010105 (PMC8444284; doi:10.15252/msb.202010105)
Supplement: Supplementary file 5 — Source Data for Figure 3 [file MSB-17-e10105-s001.zip › Figure3A_sourcedata/GSEA_3065/hallmarks_state2.GseaPreranked.1623416269049/HALLMARK_ALLOGRAFT_REJECTION.html]

Details for gene set HALLMARK\_ALLOGRAFT\_REJECTION[GSEA]

|  || Dataset | state2 |
| Phenotype | NoPhenotypeAvailable |
| Upregulated in class | na\_pos |
| GeneSet | HALLMARK\_ALLOGRAFT\_REJECTION |
| Enrichment Score (ES) | 0.31004456 |
| Normalized Enrichment Score (NES) | 0.97485787 |
| Nominal p-value | 0.4891775 |
| FDR q-value | 0.5419444 |
| FWER p-Value | 1.0 |
Table: GSEA Results Summary

  

Fig 1: Enrichment plot: HALLMARK\_ALLOGRAFT\_REJECTION      
 Profile of the Running ES Score & Positions of GeneSet Members on the Rank Ordered List

  

| PROBE | GENE SYMBOL | GENE\_TITLE | RANK IN GENE LIST | RANK METRIC SCORE | RUNNING ES | CORE ENRICHMENT || 1 | BRCA1 |  |  | 92 | 0.381 | 0.0532 | Yes |
| 2 | NME1 |  |  | 214 | 0.274 | 0.0857 | Yes |
| 3 | CCND2 |  |  | 227 | 0.263 | 0.1279 | Yes |
| 4 | UBE2N |  |  | 325 | 0.224 | 0.1547 | Yes |
| 5 | NPM1 |  |  | 351 | 0.216 | 0.1878 | Yes |
| 6 | RPS3A |  |  | 719 | 0.148 | 0.1732 | Yes |
| 7 | ABCE1 |  |  | 746 | 0.144 | 0.1943 | Yes |
| 8 | EIF5A |  |  | 801 | 0.138 | 0.2113 | Yes |
| 9 | MRPL3 |  |  | 906 | 0.128 | 0.2214 | Yes |
| 10 | EIF3A |  |  | 971 | 0.122 | 0.2347 | Yes |
| 11 | RPS9 |  |  | 1007 | 0.119 | 0.2508 | Yes |
| 12 | RPL39 |  |  | 1074 | 0.113 | 0.2624 | Yes |
| 13 | AKT1 |  |  | 1093 | 0.112 | 0.2790 | Yes |
| 14 | CCND3 |  |  | 1164 | 0.107 | 0.2892 | Yes |
| 15 | RPL9 |  |  | 1166 | 0.107 | 0.3067 | Yes |
| 16 | TPD52 |  |  | 1493 | 0.087 | 0.2864 | Yes |
| 17 | BCAT1 |  |  | 1503 | 0.086 | 0.2997 | Yes |
| 18 | IFNGR2 |  |  | 1538 | 0.085 | 0.3100 | Yes |
| 19 | RIPK2 |  |  | 1947 | 0.067 | 0.2776 | No |
| 20 | EIF3J |  |  | 2081 | 0.062 | 0.2736 | No |
| 21 | HIF1A |  |  | 2182 | 0.058 | 0.2725 | No |
| 22 | RPS19 |  |  | 2268 | 0.055 | 0.2725 | No |
| 23 | UBE2D1 |  |  | 2304 | 0.054 | 0.2776 | No |
| 24 | STAT1 |  |  | 2336 | 0.053 | 0.2830 | No |
| 25 | EIF3D |  |  | 2985 | 0.034 | 0.2196 | No |
| 26 | ETS1 |  |  | 2998 | 0.034 | 0.2239 | No |
| 27 | CSK |  |  | 3000 | 0.034 | 0.2294 | No |
| 28 | CAPG |  |  | 3281 | 0.028 | 0.2042 | No |
| 29 | MTIF2 |  |  | 3667 | 0.021 | 0.1666 | No |
| 30 | TRAF2 |  |  | 3754 | 0.020 | 0.1607 | No |
| 31 | BCL10 |  |  | 4493 | 0.007 | 0.0832 | No |
| 32 | IFNAR2 |  |  | 5074 | -0.001 | 0.0216 | No |
| 33 | APBB1 |  |  | 5288 | -0.005 | -0.0004 | No |
| 34 | MAP3K7 |  |  | 5296 | -0.005 | -0.0003 | No |
| 35 | EIF4G3 |  |  | 5437 | -0.007 | -0.0140 | No |
| 36 | GALNT1 |  |  | 6270 | -0.021 | -0.0992 | No |
| 37 | IRF7 |  |  | 6755 | -0.031 | -0.1457 | No |
| 38 | ABI1 |  |  | 6766 | -0.031 | -0.1417 | No |
| 39 | GLMN |  |  | 6904 | -0.034 | -0.1506 | No |
| 40 | TAP2 |  |  | 7075 | -0.038 | -0.1624 | No |
| 41 | NCK1 |  |  | 7576 | -0.053 | -0.2070 | No |
| 42 | ACVR2A |  |  | 7795 | -0.059 | -0.2204 | No |
| 43 | IKBKB |  |  | 7809 | -0.060 | -0.2119 | No |
| 44 | IFNGR1 |  |  | 7945 | -0.065 | -0.2156 | No |
| 45 | TAP1 |  |  | 7963 | -0.066 | -0.2065 | No |
| 46 | IL27RA |  |  | 8040 | -0.069 | -0.2033 | No |
| 47 | EGFR |  |  | 8094 | -0.071 | -0.1972 | No |
| 48 | THY1 |  |  | 8139 | -0.074 | -0.1897 | No |
| 49 | DEGS1 |  |  | 8459 | -0.093 | -0.2083 | No |
| 50 | TGFB1 |  |  | 8497 | -0.095 | -0.1965 | No |
| 51 | CSF1 |  |  | 8560 | -0.101 | -0.1865 | No |
| 52 | CD74 |  |  | 8754 | -0.117 | -0.1877 | No |
| 53 | FLNA |  |  | 8824 | -0.126 | -0.1742 | No |
| 54 | HDAC9 |  |  | 8833 | -0.127 | -0.1541 | No |
| 55 | TGFB2 |  |  | 8921 | -0.137 | -0.1406 | No |
| 56 | TAPBP |  |  | 8966 | -0.144 | -0.1215 | No |
| 57 | F2R |  |  | 9026 | -0.153 | -0.1025 | No |
| 58 | CD47 |  |  | 9265 | -0.219 | -0.0916 | No |
| 59 | B2M |  |  | 9383 | -0.331 | -0.0494 | No |
| 60 | TIMP1 |  |  | 9387 | -0.334 | 0.0056 | No |
Table: GSEA details [plain text format]

  

Fig 2: HALLMARK\_ALLOGRAFT\_REJECTION: Random ES distribution      
 Gene set null distribution of ES for **HALLMARK\_ALLOGRAFT\_REJECTION**
